# Machine Learning-Augmented Propensity Score Analysis of Percutaneous Coronary Intervention in Over 30 Million Cancer and Non-cancer Patients

**DOI:** 10.3389/fcvm.2021.620857

**Published:** 2021-04-06

**Authors:** Dominique J. Monlezun, Sean Lawless, Nicolas Palaskas, Shareez Peerbhai, Konstantinos Charitakis, Konstantinos Marmagkiolis, Juan Lopez-Mattei, Mamas Mamas, Cezar Iliescu

**Affiliations:** ^1^Department of Cardiology, The University of Texas M.D. Anderson Cancer Center, Houston, TX, United States; ^2^Division of Cardiovascular Medicine, The University of Texas Health Sciences Center at Houston, Houston, TX, United States; ^3^Premier Heart and Vascular Center, Zephyrhills, FL, United States; ^4^Keele Cardiovascular Research Group, Department of Cardiology, Royal Stroke Hospital Stoke on Trent, Stoke-on-Trent, United Kingdom

**Keywords:** PCI - percutaneous coronary intervention, cancer, cardio-oncology, onco-cardiology, disparites, machine laerning

## Abstract

**Background:** It is unknown to what extent the clinical benefits of PCI outweigh the risks and costs in patients with vs. without cancer and within each cancer type. We performed the first known nationally representative propensity score analysis of PCI mortality and cost among all eligible adult inpatients by cancer and its types.

**Methods:** This multicenter case-control study used machine learning–augmented propensity score–adjusted multivariable regression to assess the above outcomes and disparities using the 2016 nationally representative National Inpatient Sample.

**Results:** Of the 30,195,722 hospitalized patients, 15.43% had a malignancy, 3.84% underwent an inpatient PCI (of whom 11.07% had cancer and 0.07% had metastases), and 2.19% died inpatient. In fully adjusted analyses, PCI vs. medical management significantly reduced mortality for patients overall (among all adult inpatients regardless of cancer status) and specifically for cancer patients (OR 0.82, 95% CI 0.75–0.89; *p* < 0.001), mainly driven by active vs. prior malignancy, head and neck and hematological malignancies. PCI also significantly reduced cancer patients' total hospitalization costs (beta USD$ −8,668.94, 95% CI −9,553.59 to −7,784.28; *p* < 0.001) independent of length of stay. There were no significant income or disparities among PCI subjects.

**Conclusions:** Our study suggests among all eligible adult inpatients, PCI does not increase mortality or cost for cancer patients, while there may be particular benefit by cancer type. The presence or history of cancer should not preclude these patients from indicated cardiovascular care.

## Highlights

This is a nationally representative multicenter comprehensive analysis of inpatient mortality and total costs of PCI in all eligible hospitalized patients with and without cancer (including sub-group analysis by CAD, cancer by primary organ site, active vs. prior cancer, and ACS). Our analysis is the first in this population to suggest a significant and independent inpatient mortality and cost benefit for PCI vs. medical management particularly for cancer patients (shown both with propensity score adjusting for the likelihood of undergoing PCI among all inpatients and within CAD patients alone), while suggesting there may be a unique cancer and coronary artery disease interaction that is seen in our analysis with certain cancer types having more pronounced mortality benefit compared to others. This study suggests that PCI is safe for cancer patients regardless of their primary malignancy type, active or prior malignancy status, and ACS status.

## Introduction

Cardiovascular diseases and cancer are the most prevalent chronic diseases and are the leading causes of morbidity and mortality in the world; specifically, one in six deaths and an estimated total of 9.6 million deaths in 2018 were attributable to cancer (1–3). Cardiovascular diseases and several cancer types share similar modifiable risk factors: high body mass index, low fruit and vegetable intake, lack of physical activity, and tobacco and alcohol use (4–7). Cancer itself is a pro-inflammatory and hypercoagulable state that increases the risk of cardiovascular events (4, 8–14). Certain primary malignancies are more likely than others to be associated with CAD, either due to shared risk factors or because their required treatments are associated with accelerated atherosclerosis (4, 5, 15–17). Aside from the clinical impact, the economic impact of cancer also is increasing with the United States' annual direct medical costs (i.e., the total of all healthcare expenditures) for cancer totaled over $80 billion (7, 18).

Further cancer patients with comorbid CAD are less likely to be treated with percutaneous coronary intervention (PCI) compared with the general population (9, 19) as they present with higher risk of complications from PCI and increased frailty (20–24). This risk is more pronounced in specific primary malignancies (i.e., lung cancer) and with the presence of metastases (20). With improved patient survival from novel cancer treatments, as well as the parallel increase in the safety of interventional procedures, the use of PCI in patients with comorbid cancer has recently been revisited (9, 20, 21, 25–32). This recent Nationwide Inpatient Sample offers an opportunity to evaluate the impact of current (with and without metastatic disease) or historical cancer diagnosis on clinical and economical outcomes (cost and length of stay). We sought therefore to conduct the first nationally representative analysis of PCI vs. no PCI among all CAD inpatients with and without cancer and among all available cancer types for mortality and cost using machine learning-augmented propensity score analysis including with racial and income disparity analysis.

## Methods

### Study Design

We sought to conduct the first nationally representative analysis of PCI vs. no PCI among all CAD inpatients with and without cancer and among all available cancer types for mortality and cost using machine learning-augmented propensity score analysis including with racial and income disparity analysis This study is thus a multi-center analysis of inpatient mortality (primary endpoint) and total costs (secondary endpoint) among all eligible hospitalized adults; it assessed the association among the endpoints and PCI (yes/no) for acute coronary syndrome (ACS, including unstable angina/including non-ST segment elevation myocardial infarction [UA/NSTEMI] and STEMI) and PCI and cancer (yes/no overall, including overall and comparatively by primary organ site). To reduce confounding bias in this non-randomized studies, the above endpoints were assessed in the above sub-group stratified analyses to facilitate result interpretation. The 2016 NIS dataset was selected for this study because it is the latest and best reflects current clinical trends in PCI use. Study inclusion criteria were all NIS hospitalizations for adults 18 years or older during 2016. This study used de-identified data and was conducted according to the ethical principles in the Declaration of Helsinki.

Subjects undergoing PCI were identified by the ICD-10 procedure codes of 00.66 (percutaneous transluminal coronary angioplasty), 36.06 [insertion of non-drug-eluting coronary artery stent(s)], or 36.07 [insertion of drug-eluting coronary artery stent(s)]. HCUP tools such as the Clinical Classification Software, which had been used prior to the NIS 2016 dataset for such purposes as classifying cancer (e.g., by primary type, current vs. historical), were not used in this study because they were found by HCUP as a beta version to be unreliable when applied to the 2016 dataset's ICD-10 data.

### Data Source

The data source for this study was the 2016 NIS for hospital discharges. The NIS is largest all-payer inpatient dataset in the nation, sponsored by the US Department of Health and Human Services' Agency for Healthcare Research and Quality and maintained within the Healthcare Cost and Utilization Project (HCUP). The NIS began in 2004 with data collection from select hospitals and expanded in 2012 to encompass discharge data from all HCUP participating hospitals. In 2016, the NIS data coding adopted the International Classification of Diseases, Tenth Revision, Clinical Modification (ICD-10-CM). The NIS currently accounts for ~1 in 5 discharges from all community hospitals in the United States. To reduce sampling bias, the sampling strategy has been modified in the most recent data to produce results more generalizable to all inpatient discharges in the country and so the associated sampling weights were applied to this analysis.

### Statistical Analysis

Descriptive statistics for demographics (i.e., age, sex, race, insurance) and comorbidities were performed for the full sample. Comorbidities were selected for analysis (and identified in the dataset by their ICD-10 scores) on the basis of their clinical and/or statistical significance for similar studies in the existing literature. The comorbidities included in this study were diabetes, hypertension, peripheral vascular disease, hyperlipidemia, smoking, obesity, poor diet, stroke, congestive heart failure, cardiac arrest, myocardial infarction, cardiogenic shock, valvular disease, anemia, chronic obstructive pulmonary disease, coagulopathy, chronic kidney disease, and malignancy (overall and by primary malignancy type).

Bivariable analysis was then conducted separately according to the following: (a) inpatient mortality (yes/no); (b) PCI (yes/no) among the overall sample, stratified by metastases (yes/no) and in subgroup analyses among patients with malignancy; (c) PCI vessel number (multi- vs. single-vessel); (d) malignancy (yes/no) in subgroup analyses among patients who died with UA/NSTEMI and separately among those with STEMI; (e) length of stay by primary malignancy type; (f) total cost by primary malignancy type. For continuous variables, independent sample *t*-tests were performed to compare means and Wilcoxon rank sum tests were performed for medians. For categorical variables, Pearson chi square tests or Fisher exact tests were performed to compare proportions.

Variables found to be statistically significant in the bivariable analysis were then included in forward and backward stepwise regression to augment decision-making on which variables should be included in the final multivariable regression models. This regression analysis was conducted to assess the following outcomes: (a) inpatient mortality (by logistic) and, (b) total hospital costs (by linear, adjusting with the additional variable of length of stay). The regression models separately assessed these outcomes according to the following major predictors: (a) historical or active malignancy (yes/no), and primary malignancy type (brain and nervous system, head or neck, thyroid, breast, lung, esophagus, stomach, pancreas, liver or bile system, rectum or anus, colon, peritoneum, bone or connective tissue system, hematological malignancies [including Hodgkin lymphoma, Non-Hodgkin lymphoma, leukemia, and multiple myeloma], skin, uterus, cervix, ovarian, prostate, testes, bladder, and renal). Sub-group analysis without propensity score adjustment was conducted separately according to history of CAD (additionally with stratified analysis by ACS and active or prior malignancy), active malignancy, prior malignancy, presenting diagnosis of ACS, UA/NSTEMI, and STEMI. These models featured the interaction between PCI and malignancy, while adjusting for age, race, income, metastases, and mortality risk by DRG (other variables were excluded based upon the below machine learning analysis and diagnostic testing to produce the most clinically and statistically justifiable models).

Next, machine learning–backed propensity score–adjusted multivariable regression was conducted for mortality and controlled for age, race, income, presence of metastases, and mortality risk by diagnosis-related group in addition to the likelihood of undergoing PCI and the NIS weights accounting for the cluster sample data structure. The propensity score was then created for the likelihood of undergoing PCI (the treatment), balance was confirmed among blocks, and then the propensity score was included in the final regression models as an adjusted variable. This causal inference approach (propensity score adjustment) was selected because it is a widely accepted methodology to reduce but not eliminate selection bias and the effect of confounding variables. Such competing causal inference approaches as fixed, random, and mixed effects were not appropriate (though these have the added advantage of reducing unobserved variable bias) because the dataset lacked adequate repeated hospitalizations from the same subjects. Propensity score adjustment was used rather than covariate adjustment without the propensity score to enable a more complicated propensity score model (i.e., able to test interactions and higher order terms to produce the most robust estimated probability of treatment assignment) without risking over-parameterizing while still permitting diagnostic analysis of the final models to be done to confirm superior performance to simple covariate adjustment without the propensity score. Finally, propensity score adjustment rather than competing propensity score techniques was used because of its superior performance in the appropriate context (confirmed by current statistical theory and adequate diagnostic quantitative testing of the final models in cardiovascular studies) (33, 34).

The utility of this above hybrid analytic approach, which integrates the traditional statistical method of frequentist-based multivariable regression (supported by propensity score-based causal inference analysis) and supervised learning-based machine learning has been previously demonstrated, as causal inference results which are more familiar to medical science audiences can be confirmed and replicated automatically through machine learning (and thus may accelerate real-time findings on larger high-dimensional datasets as they already increasingly do for other economic sectors outside of medicine), while producing more rapid and accurate results compared to traditional statistics (35–40).

To modify the final models until optimal performance was achieved, performance was first assessed relative to results from backward propagation neural network machine learning to ensure comparability by root mean squared error and accuracy. Regression model performance was additionally assessed with correlation matrix, area under the curve, Hosmer-Lemeshow goodness-of-fit test, Akaike and Schwarz Bayesian information criterion, variance inflation factor, and tolerance, multicollinearity, and specification error. An academic physician-data scientist and biostatistician confirmed that the final regression models were sufficiently supported by the existing literature and clinical and statistical theory. Fully adjusted regression results were reported with 95% confidence intervals (CIs) with statistical significance set at a 2-tailed *p* < 0.05. Statistical analysis was performed with STATA 14.2 (STATA Corp, College Station, Texas, USA), and machine learning analysis was performed with Java 9 (Oracle, Redwood Chores, California, USA).

## Results

### Overall Sample Descriptive and Bivariable Analyses

Among the 30,195,722 hospitalized patients meeting study criteria, the mean (SD) age was 57.51 (20.33) years; 17,558,812 (58.15%) were female; 21,043,399 (67.69%) were Caucasian; 4,659,200 (15.43%) had cancer (of whom 2,117,606 [45.45%] was active); 1,159,516 (3.84%) underwent an inpatient PCI, and 661,286 (2.19%) died that hospitalization (Table 1). Among all hospitalized patients, 19.45% had CAD, 2.67% had UA/NSTEMI, and 0.75% had STEMI. The most common primary malignancies in patients in whom PCI was performed were prostate (2.34%), breast (1.83%), skin (1.74%), gastrointestinal (1.54%), and hematological (1.48%) cancers.

**Table 1:** Descriptive statistics by common primary malignancies and bivariable analysis by cancer (*N* = 30,195,722).

**Variables**	**Sample**	Cancer^*^		Breast^*^	Lung^*^	Colon^*^	Prostate^*^	Hematological^*^	Skin^*^
	***N*** **= 30,195,722**	**No (*n* = 25,535,129)**	**Yes (*n* = 4,660,593)**	***n*** **= 751,105**	***n*** **= 566,434**	***n*** **= 442,755**	***n*** **= 637,100**	***n*** **= 660,260**	***n*** **= 500,445**
**Demographics, no. (%)**									
Age, mean (SD)	57.51 (20.33)	55.46 (0.01)	68.70 (0.01)	70.33 (13.76)	69.77 (10.84)	71.59 (13.62)	75.28 (0.03)	65.97 (0.4)	73.43 (12.60)
Female	17,558,812 (58.15)	15,203,616 (59.54)	2,353,133 (50.49)	743,068 (98.93)	279,875 (49.41)	218,987 (49.46)	0 (0.00)	294,212 (44.56)	217,143 (43.39)
Race									
**All groups**									
White	20,469,680 (67.79)	16,909,362 (66.22)	3,560,693 (76.40)	571,290 (76.06)	449,012 (79.27)	332,863 (75.18)	478,207 (75.06)	490,045 (74.22)	475,273 (94.97)
Black	4,568,613 (15.13)	16,909,362 (15.80)	535,968 (11.50)	97,118 (12.93)	66,726 (11.78)	55,344 (12.50)	96,521 (15.15)	75,864 (11.49)	4,704 (0.94)
Hispanic	3,273,216 (10.84)	2,949,307 (11.55)	322,047 (6.91)	46,644 (6.21)	24,413 (4.31)	31,878 (7.20)	36,824 (5.78)	54,736 (8.29)	11,010 (2.20)
Asian	812,265 (2.69)	704,770 (2.76)	108,592 (2.33)	16,524 (2.20)	12,971 (2.29)	10715 (2.42)	10,002 (1.57)	16,176 (2.45)	1,702 (0.34)
Native American	187,213 (0.62)	168,532 (0.66)	17,244 (0.37)	2,554 (0.34)	1,926 (0.34)	1,727 (0.39)	1,529 (0.24)	2,311 (0.35)	751 (0.15)
Other	884,735 (2.93)	768,607 (3.01)	115,583 (2.48)	16,975 (2.26)	11,329 (2.00)	10,183 (2.30)	14016 (2.20)	21,194 (3.21)	7,006 (1.40)
**Insurance**									
All groups									
Commercial	8,343,078 (27.63)	7,292,833 (28.56)	1,051,430 (22.56)	161,112 (21.45)	101,958 (18.00)	84,743 (19.14)	103,465 (16.24)	170,149 (25.77)	92,532 (18.49)
Medicare	14,167,833 (46.92)	11,123,102 (43.56)	3,043,367 (65.30)	518,788 (69.07)	392,142 (69.23)	312,231 (70.52)	497639 (78.11)	403,353 (61.09)	379,187 (75.77)
Medicaid	5,622,443 (18.62)	5,232,148 (20.49)	392,888 (8.43)	52,577 (7.00)	50,186 (8.86)	30,727 (6.94)	17,329 (2.72)	60,216 (9.12)	15,764 (3.15)
VA	887,754 (2.94)	789,035 (3.09)	102,067 (2.19)	11,342 (1.51)	14,161 (2.50)	8,545 (1.93)	13,889 (2.18)	15,252 (2.31)	8,808 (1.76)
None	1,171,594 (3.88)	1,100,564 (4.31)	70,375 (1.51)	7,286 (0.97)	7,873 (1.39)	6,508 (1.47)	4,715 (0.74)	11,356 (1.72)	4,154 (0.83)
**Medical history**									
Diabetes	5,703,972 (18.89)	4,739,320 (18.56)	964,277 (20.69)	153,826 (20.48)	106,603 (18.82)	100,505 (22.70)	144,494 (22.68)	126,968 (19.23)	98,137 (19.61)
Hypertension	16,405,336 (54.33)	13,347,212 (52.27)	3,055,951 (65.57)	506,846 (67.48)	369,768 (65.28)	302,933 (68.42)	478,781 (75.15)	394,703 (59.78)	364,024 (72.74)
PVD	1,105,163 (3.66)	947,353 (3.71)	218,582 (4.69)	26,814 (3.57)	40,160 (7.09)	21,252 (4.80)	38,417 (6.03)	24,033 (3.64)	30,677 (6.13)
HLD	9508632.8578 (31.49)	7,673,306 (30.05)	1,834,875 (39.37)	302,620 (40.29)	227,140 (40.10)	172,896 (39.05)	320,334 (50.28)	224,026 (33.93)	253,575 (50.67)
Smoking	673,365 (2.23)	620,504 (2.43)	51,267 (1.10)	5,483 (0.73)	10,252 (1.81)	4,073 (0.92)	5,798 (0.91)	5,810 (0.88)	3,853 (0.77)
Obesity	4,399,517 (14.57)	3,878,786 (15.19)	520,588 (11.17)	98,921 (13.17)	41,973 (7.41)	48,216 (10.89)	58,868 (9.24)	62,989 (9.54)	60,254 (12.04)
Poor diet	27,176 (0.09)	56,177 (0.22)	6,059 (0.13)	1,052 (0.14)	623 (0.11)	531 (0.12)	956 (0.15)	858 (0.13)	751 (0.15)
CVA/TIA	1,295,396 (4.29)	1,090,350 (4.27)	195,745 (4.42)	35,602 (4.74)	26,226 (4.63)	17,267 (3.90)	36,315 (5.70)	23,571 (3.57)	32,929 (6.58)
CHF	1,669,823 (5.53)	1,371,236 (5.37)	298,278 (6.40)	49,798 (6.63)	38,744 (6.84)	30,417 (6.87)	49,120 (7.71)	46,812 (7.09)	37,684 (7.53)
HFrEF	766,971 (2.54)	633,271 (2.48)	132,827 (2.85)	18,102 (2.41)	17,050 (3.01)	13,548 (3.06)	25,611 (4.02)	21,591 (3.27)	16,365 (3.27)
Cardiac arrest	238,546 (0.79)	201,728 (0.79)	37,285 (0.80)	4,732 (0.63)	6,401 (1.13)	3,365 (0.76)	5,033 (0.79)	6,140 (0.93)	3,153 (0.63)
	***N*** **= 30,195,722**	**No (*n* = 25,535,129)**	**Yes (*n* = 4,660,593)**	***n*** **= 751,105**	***n*** **= 566,434**	***n*** **= 442,755**	***n*** **= 637,100**	***n*** **= 660,260**	***n*** **= 500,445**
Myocardial infarction	1,008,537 (3.34)	893,730 (3.50)	135,157 (2.90)	20,881 (2.78)	16,710 (2.95)	12,973 (2.93)	26,503 (4.16)	17,629 (2.67)	18,116 (3.62)
STEMI	226,468 (0.75)	204,281 (0.80)	22,837 (0.49)	3,455 (0.46)	2,832 (0.50)	2,125 (0.48)	4,778 (0.75)	2,839 (0.43)	2,903 (0.58)
UA/NSTEMI	806,226 (2.67)	692,002 (2.71)	112,786 (2.42)	17,501 (2.33)	13,934 (2.46)	10,892 (2.46)	21,789 (3.42)	14,922 (2.26)	15,214 (3.04)
Cardiogenic shock	135,881 (0.45)	120,015 (0.47)	16,778 (0.36)	2,479 (0.33)	2,266 (0.40)	1,461 (0.33)	2,931 (0.46)	3,301 (0.50)	1,802 (0.36)
Valvular disease	1,603,393 (5.31)	1,297,184 (5.08)	307,599 (6.60)	60,689 (8.08)	29,228 (5.16)	30,107 (6.80)	53,771 (8.44)	45,756 (6.93)	50,195 (10.03)
Anemia	6,147,849 (20.36)	4,716,338 (18.47)	1,431,734 (30.72)	191,832 (25.54)	176,048 (31.08)	146,685 (33.13)	173,355 (27.21)	306,559 (46.43)	115,252 (23.03)
COPD	4,924,922 (16.31)	3,919,642 (15.35)	1,006,222 (21.59)	137,753 (18.34)	306,611 (54.13)	80,404 (18.16)	122,705 (19.26)	106,566 (16.14)	101,290 (20.24)
Coagulation disorder	1,890,252 (6.26)	1,452,949 (5.69)	438,562 (9.41)	51,376 (6.84)	47,977 (8.47)	32,410 (7.32)	52,370 (8.22)	119,837 (18.15)	39,936 (7.98)
CKD 3-5	3,427,214 (11.35)	2,801,204 (10.97)	625,452 (13.42)	84,875 (11.30)	54,887 (9.69)	62,960 (14.22)	114,423 (17.96)	99,699 (15.10)	76,818 (15.35)
ESRD	1,081,007 (3.58)	947,353 (3.71)	134,691 (2.89)	16,825 (2.24)	9,176 (1.62)	12,397 (2.80)	20,897 (3.28)	24,430 (3.70)	12,161 (2.43)

*SD, standard deviation; VA, Veteran Affairs; PVD, peripheral vascular disease; HLD, hyperlipidemia; CVA, cerebrovascular disease; TIA, transient ischemia attack; CHF, congestive heart failure; HFrEF, heart failure with reduced ejection fraction; STEMI, ST segment elevation myocardial infarction, NSTEMI, non-ST segment elevation myocardial infarction; UA, unstable angina; COPD, chronic obstructive pulmonary disease; CKD, chronic kidney disease; ESRD, end stage renal disease; PCI, percutaneous coronary intervention; CABG, coronary artery bypass graft*.

* *p < 0.05*.

### PCI Sub-group Bivariable Analyses

Among PCI patients, 11.07% had cancer, 0.07% had metastatic disease and he top primary malignancies in which multi- vs. single-vessel PCI was performed at significantly higher proportion compared with other malignancies included breast (2.09%, *p* < 0.001), hematological (1.66%, *p* < 0.001), gastrointestinal (1.66%, *p* < 0.001), colon (1.02%, *p* = 0.001), and lung cancers (1.02%, *p* < 0.001).

There was notable mortality, cost, and length of stay differences according to primary malignancy type, active vs. prior malignancy, and metastasis (Table 2). Among PCI patients, the highest mortality by primary malignancy was for prostate (14.87%), lung (14.27%), breast (10.88%), and skin (10.88%) cancers. There was significantly higher mortality in cancer vs. non-cancer patients with NSTEMI/UA (9.34 vs. 6.78%, *p* < 0.001) and STEMI (17.70 vs. 10.83%, *p* < 0.001). Among PCI patients grouped by primary malignancy, the highest mean length of stay (in days) was for bone/connective tissue (11.5, SD 19.25), liver/bile (9.73, SD 11.53), and pancreatic cancers (9.45, SD 10.12; *p* < 0.001), and the highest mean costs were for liver/biliary cancer ($187,742, SD 308,824.00), bone/connective tissue cancer ($164,922.70, SD 223,373.20), and leukemia ($142,577.30, SD 179,511.40; *p* < 0.001).

**Table 2:** Bivariable mortality analysis by myocardial infarction and percutaneous coronary intervention (*N* = 30,195,722).

**Ariables (%)**	**Mortality: 2,448,873 (8.11%)**						**Cost, median United States dollars ($) 29,143 (range 15,587–56,287)**						**Length of stay, median days 3 (range 2–5)**					
	**UA/NSTEMI: 2,155,975 (7.14%)**			**STEMI: 3,478,547 (11.52%)**			**UA/NSTEMI: $56,059 (29,194–106,095)**			**STEMI: $74,861 (46,585–122,465)**			**UA/NSTEMI: 4 (2–7)**			**STEMI: 3 (2–5)**		
	**No PCI: 1,814,469 (84.16%)**	**PCI: 341,506 (15.84%)**	***P*-value**	**No PCI: 1,946,595 (55.96%)**	**PCI: 1,531,952 (44.04%)**	***P*-Value**	**No PCI: $39,618 (20,445–83,435)**	**PCI: $72,680 (44,663–123,811)**	***P*-value**	**No PCI: $38,008 (18,144–89,103)**	**PCI: $81,960 (55,957–128,357)**	***P*-value**	**No PCI: 4 (2–8)**	**PCI: 3 (2–7)**	***P*-value**	**No PCI: 4 (2–8)**	**PCI: 3 (2–4)**	***P*-value**
No cancer	11.30	2.35	<0.001	26.75	6.41	<0.001	39,483 (20,232–83,797)	72,608 (44,549–123,803)	<0.001	37,676 (17,780–90,558)	81,959 (55,892–128,242)	<0.001	4 (2–8)	3 (2–6)	<0.001	4 (2–8)	3 (2–4)	<0.001
Cancer	13.32	3.02	<0.001	32.12	8.90	<0.001	40,406 (21,485–81,527)	73,137 (45,496–123,817)	<0.001	39,688 (20,153–81,272)	81,989 (56,660–129,395)	<0.001	5 (3–8)	4 (2–7)	<0.001	4 (2–8)	3 (2–5)	<0.001
Breast																		
History	9.84	1.73	<0.001	27.57	8.24	<0.001	37,036 (20,468–64,145)	67,733 (41,300–111,708)	<0.001	33,348 (15,839–70,403)	80,544 (57,881–119,920)	<0.001	4 (2–7)	3 (2–6)	<0.001	4 (2–7)	3 (2–5)	0.062
Active	14.52	0.79	<0.001	39.47	16.67	0.030	39,795 (20,321–76,507)	73,325 (46,953–111,954)	<0.001	52,914 (27,204–118,261)	77,385 (51,906–118,105)	0.100	5 (2–8)	4 (2–7)	0.095	5 (3–12)	3 (2–5)	0.013
Metastatic	19.76	1.64	<0.001	42.86	28.57	0.260	37,059 (22,154–74,875)	85,280 (38,977–128,409)	<0.001	46,331 (24,845–113,660)	81,841 (49,000–135,190)	0.020	5 (2–8)	4 (2–7)	0.269	5 (3–11)	4 (2–8)	0.345
Lung						<0.001			<0.001									
History	11.30	3.57	<0.001	31.58	11.39	<0.001	38,672 (21,271–74,078)	72,994 (47,883–112,613)	<0.001	34,548 (20,597–66,873)	80,058 (57,488–135,796)	<0.001	5 (3–8)	4 (2–6)	0.006	4 (2–7)	3 (2–6)	0.549
Active	22.82	9.13	<0.001	39.59	21.19	<0.001	47,512 (24,626–91,404)	74,196 (49,782–116,071)	<0.001	40,498 (19,516–78,998)	99,581 (57,526–163,505)	<0.001	5 (3–9)	5 (3–9)	0.755	4 (2–8)	4 (2–9)	0.491
Metastatic	23.27	8.16	0.001	36.80	25.86	0.001	47,053 (24,626–88,410)	77,798 (54,411–127,294)	<0.001	38,647 (16,107–75,284)	97,537 (51,849–134,818)	<0.001	5 (3–9)	5 (3–8)	0.558	4 (2–8)	4 (2–8)	0.812
Prostate						0.144			<0.001									
History	10.16	2.32	<0.001	27.88	5.89	<0.001	34,605 (19,043–68,583)	74,589 (45,658–126,691)	<0.001	34,537 (16,899–67,835)	79,883 (54,925–126,777)	<0.001	4 (2–7)	3 (2–6)	<0.001	4 (2–7)	3 (2–5)	0.004
Active	13.59	3.45	<0.001	34.67	8.08	<0.001	39,712 (23,881–81,389)	85,479 (50,212–142,427)	<0.001	40,704 (24,864–84,969)	79,438 (55,561–137,324)	<0.001	5 (3–9)	4 (2–8)	0.007	6 (2–10)	3 (2–7)	0.004
Metastatic	14.84	4.08	0.004	31.03	18.75	<0.001	43,516 (25,273–79,595)	76,375 (47,744–137,167)	<0.001	32,685 (23,843–70,702)	67,627 (55,648–117,526)	<0.001	5 (3–9)	4 (2–9)	0.060	4 (2–9)	3 (2–6)	0.272
Colon						<0.001												
History	11.39	2.53	<0.001	27.21	8.50	<0.001	37,129 (20,418–72,128)	72,120 (44,556–114,526)	<0.001	35,246 (15,955–64,534)	81,350 (55,422–112,388)	<0.001	4 (3–8)	4 (2–7)	<0.001	4 (2–7)	3 (2–5)	0.003
Active	18.58	6.06	0.003	30.00	19.35	0.288	76,802 (35,376–148,370)	104,675 (55,870–183,304)	0.002	50,822 (23,120–108,955)	113,139 (64,982–175,087)	0.005	8 (4–13)	7 (3–12)	0.070	5 (2–12)	5 (2–15)	0.643
Metastatic	18.23	6.12	0.037	32.43	8.70	0.035	51,511 (26,892–112,133)	86,090 (50,970–174,804)	0.005	49,372 (23,120–78,839)	82,888 (64,982–175,087)	0.002	6 (3–11)	6 (2–10)	0.482	5 (3–9)	4 (2–14)	0.789
Skin									<0.001									
History	8.80	2.73	<0.001	29.66	6.73	<0.001	31832 (17,940–61,004)	69,272 (43,558–110,059)	<0.001	36,652 (17,940–73,450)	77,655 (53,331–117,858)	<0.001	4 (2–7)	3 (2–6)	<0.001	4 (1–7)	3 (2–4)	0.115
Active	15.69	5.19	0.022	18.75	14.29	0.715	46,991 (21,979–91,548)	84,049 (41,616–133,584)	<0.001	41,314 (27,015–75,622)	87,643 (53,654–125,188)	0.010	5 (3–10)	4 (2–7)	0.012	5 (3–6)	2 (2–4)	0.188
Metastatic	20.65	21.74	0.909	33.33	0.00	0.052	46,493 (21,381–83,185)	97,682 (73,386–148,753)	<0.001	59,576 (30,926–79,217)	91,565 (54,585–136,663)	0.101	5 (2–8)	7 (4–9)	0.175	6 (3–10)	5 (3–8)	0.589
Hematologic									<0.001									
History	11.58	3.45	<0.001	18.75	7.10	0.011	36,401 (19,411–70,237)	65,043 (41,059–121,736)	<0.001	40,818 (19,449–81,060)	81,527 (53,788–121,246)	<0.001	4 (2–7)	3 (2–6)	0.002	4 (3–7)	3 (2–5)	0.004
Active	18.07	7.69	<0.001	44.83	10.47	<0.001	51,713 (26,013–118,492)	85,932 (49,276–159,541)	<0.001	53,261 (24,470–147,175)	99,786 (63,124–162,734)	<0.001	5 (3–11)	5 (2–9)	0.001	5 (2–13)	3 (2–7)	0.099
Metastatic	21.35	11.76	0.364	36.84	33.33	0.907	63,282 (29,464–135,134)	81,999 (42,492–148,753)	0.18	57,441 (36,396–128,357)	59,088 (37,016–297,917)	0.738	6 (3–12)	6 (5–10)	0.330	5 (3–11)	3 (3–21)	0.962

*STEMI, ST segment elevation myocardial infarction, NSTEMI, non-ST segment elevation myocardial infarction; UA, unstable angina*.

### Overall Sample Multivariable Regression Analyses by PCI

In machine learning-backed multivariable regression fully adjusted for age, race, income, metastases, and mortality risk by DRG, PCI was associated with a significantly reduced odds of mortality for all patients among all adult inpatients regardless of cancer status (OR 0.77, 95%CI 0.75–0.79; *p* < 0.001) and specifically for cancer patients (OR 0.82, 95%CI 0.75–0.89; *p* < 0.001). This was confirmed by propensity score adjustment while significantly reducing their total hospital costs (beta USD$ −8,668.94, 95%CI −9,553.59 to −7,784.28; *p* < 0.001) independent of the length of stay.

### CAD and Active Cancer Sub-group Multivariable Regression Analyses by PCI

**Figure 1:**
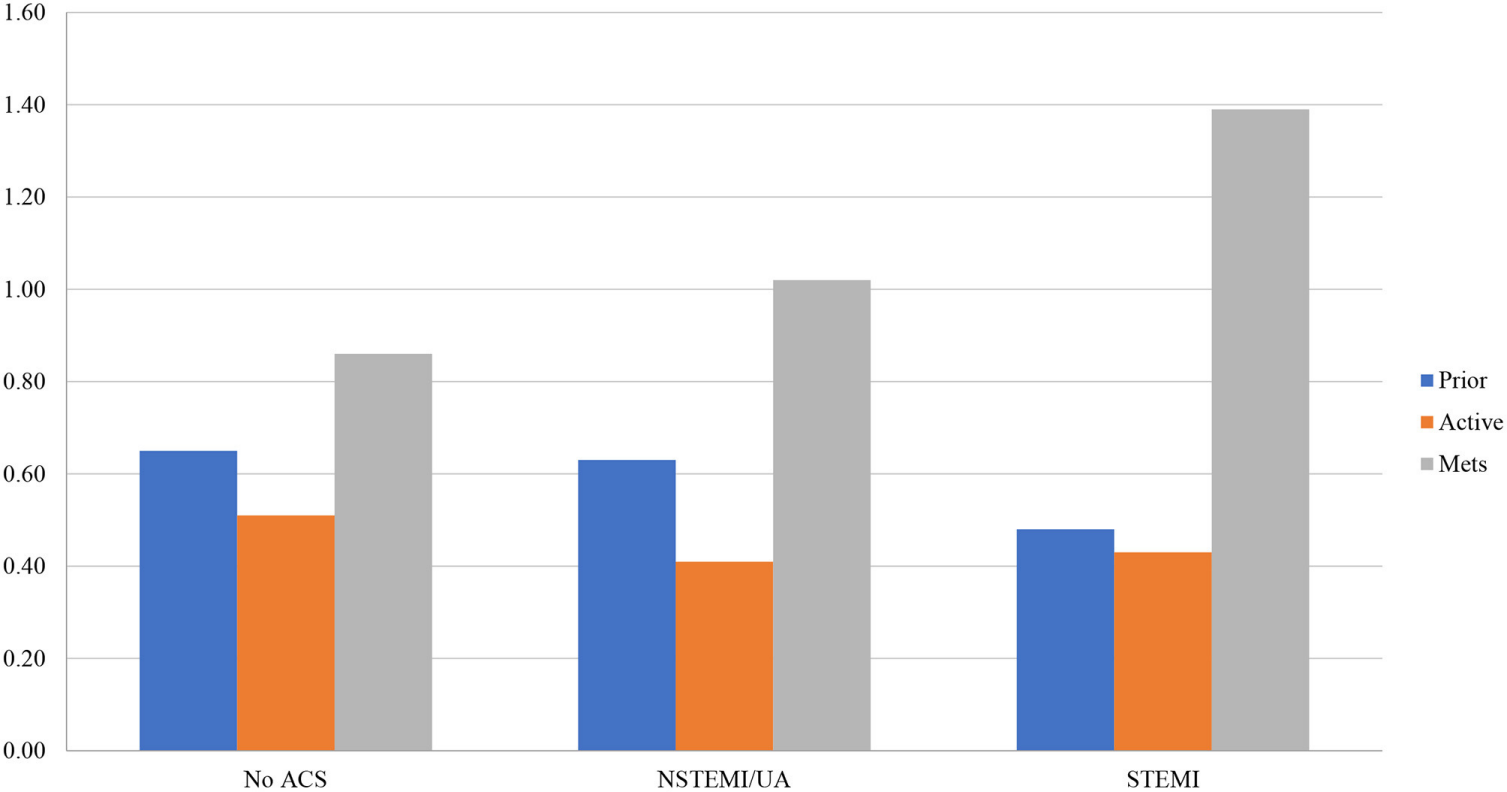
Propensity score-fully adjusted mortality odds ratios by ACS and active or prior malignancy (*N* = 30,195,722). Fully adjusted for age, race, income, metastases, and mortality risk by Disease Related Group; mets, metastasis; ACS, acute coronary syndrome, NSTEMI, non-ST segment myocardial infarction; UA, unstable angina; STEMI, ST segment myocardial infarction.

Results were similar in sub-group analysis among CAD patients and separately in prior and active cancer patients (with greater mortality reductions in patients with active [OR 0.63, 95%CI 0.56–0.71; *p* < 0.001] rather than prior malignancies [OR 0.72, 95%CI 0.65–0.79; *p* < 0.001]) (Figure 1). In the CAD sub-group with stratified analysis by ACS (UA/NSTEMI and STEMI) and active or prior malignancy, PCI vs. medical management significantly reduced mortality for all patient groups (Figure 1). Active vs. prior malignancy had mortality reductions across no ACS, UA/NSTEMI, and STEMI groups. The greatest mortality reductions among all groups were patients with active malignancy and UA/NSTEMI (OR 0.41, 95%CI 0.26–0.65; *p* < 0.001) and active malignancy with STEMI (OR 0.43, 95%CI 0.31–0.59; *p* < 0.001).

### Primary Malignancy Sub-group Multivariable Regression Analyses by PCI

**Figure 2:**
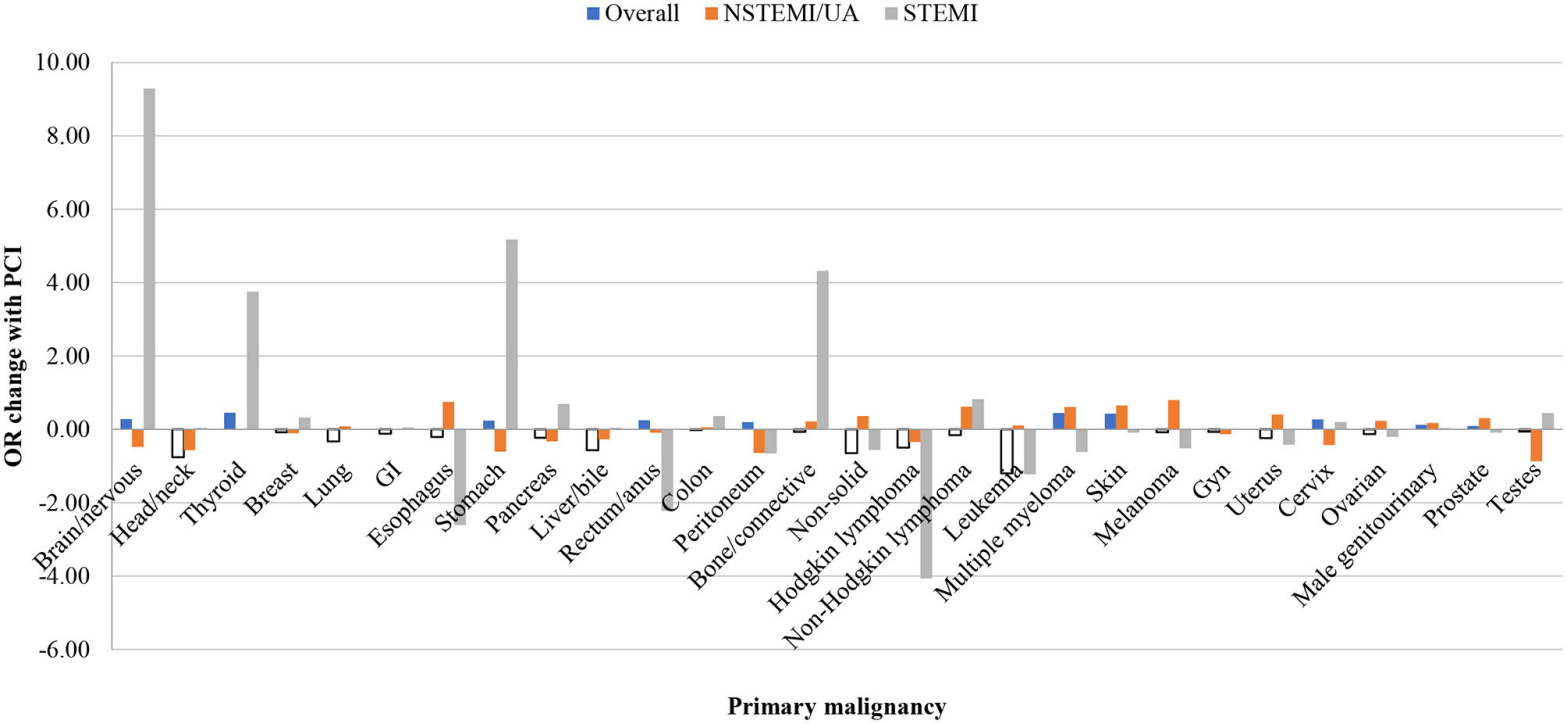
Propensity score adjusted inpatient mortality odds ratio change with ACS and PCI by primary malignancy in fully adjusted multivariable regression (*N* = 30,195,722). Multivariable regression fully adjusted for age, race, income, metastases, and mortality risk by Diagnosis Related Group (DRG); ACS, acute coronary syndrome, NSTEMI, non-ST segment myocardial infarction; UA, unstable angina; STEMI, ST segment myocardial infarction; PCI, percutaneous coronary intervention); GI, gastrointestinal; gyn, gynecological.

In sub-group analysis by primary malignancy among those with cancer, PCI was associated with a significantly reduced odds of mortality only in patients with head and neck vs. non-head and neck cancers (OR 0.34, 95%CI 0.17–0.66; *p* = 0.002), Hodgkin lymphoma vs. cancers other than Hodgkin lymphoma (OR 0.35, 95%CI 0.14–0.87; *p* = 0.025), and leukemia vs. cancers other than leukemia (OR 0.64, 95%CI 0.48–0.86; *p* = 0.003) (Figure 2). PCI in cancer patients with metastatic disease was associated with reduced mortality but not significantly (OR 0.86, 95%CI 0.71–1.04; *p* = 0.110). Similarly, PCI also was associated with non-significantly reduced mortality in patients with non-solid vs. solid tumors (OR 0.85, 95%CI 0.71–1.02; *p* = 0.079). There were no significant disparities by income or race among PCI subjects.

## Discussion

This propensity score adjusted nationally representative analysis of over 30 million hospitalized adults suggests that PCI does not increase inpatient mortality (primary endpoint) nor total costs (secondary endpoint) among patients with cancer regardless of whether they had concurrent non-ACS, UA/NSTEMI, or STEMI indications (with particular primary malignancies driving more of the above associations than others). These results may support offering PCI when deemed appropriate by clinicians to cancer patients who have traditionally been excluded from or underrepresented in cardiovascular randomized trials (which may account for some of the current hesitation with considering more readily such invasive treatment options). The above clinical findings may thus allow more informed clinician-patient discussions about treatment options at a time when such cardio-oncology patients with both CAD and cancer represent a sizeable and growing portion of the PCI patient population nationally (as this analysis of over 1 million PCI procedures detected more than 1 in 10 being performed in such patients with both cancer and heart disease).

The most common primary malignancies nationally per our study were prostate, gastrointestinal, breast, skin cancers, lung and hematological. Prostate and skin cancers were the most common primary malignancies in which single-vessel PCI was performed as they can be viewed as more favorable PCI candidates, and were clinical practice is often parallel to non-cancer patients. Conversely, patients with lung, breast, gastrointestinal, and hematological cancers are the cancer patient sub-groups in which multivessel PCI was performed at a higher proportion than single-vessel PCI probably due to time constraints, taking advantage of the window of opportunity and complete revascularization. Also, in lung cancer patients the additional CAD burden can be explained by the higher prevalence of cardiovascular risk factors (such as smoking) and the cancer treatments that promote early atherosclerosis (including radiation therapy) (4, 5, 15–17). Prior studies of NIS data, as well as our analysis, have shown that PCI in the setting of lung cancer was associated with a higher risk of inpatient mortality when compared to other primary malignancies (20). The short interval to initiation of cancer treatment due to the aggressive nature of majority of these tumors could be utilized for a more comprehensive cardiovascular risk stratification/evaluation and to optimize medical management in an attempt to minimize cardiovascular morbidity and mortality.

VEGF inhibitors (bevacizumab, sunitinib, sorafenib, pazopanib), novel immunotherapies can be associated with vascular toxicity, enhanced inflammation of atherosclerotic plaques, destabilization of pre-existing plaques, and promotion of plaque rupture (41–47). Our study provides an overall picture of the impact of such cancer treatments, but the lack of data granularity prohibits more rigorous understanding of the impact of cancer treatments on CAD burden and PCI outcomes. Regardless, our results are consistent with prior studies that support the safety and efficacy of PCI in cancer patient (9, 21, 25, 28, 30).

The primary organ site and stage including presence or absence of metastatic disease are the main driver of outcomes in a cancer patient population. Metastatic patients have higher risk for inpatient mortality probably due to the greater extent of their oncologic disease. In our analysis, 1 in 20 cancer patients who underwent PCI had metastatic disease, and the intervention still appeared to reduce mortality.

Additionally our analysis also demonstrated that cancer patients who received PCI had decreased total hospital costs of ~$8,000–9,000, independent of their inpatient length of stay, clinical acuity, mortality risk (as calculated by DRGs), and other factors rigorously tested in propensity score adjustment. The inherent cost of the procedure could potentially be reduced by their immediate symptomatic improvement and therefore decreased laboratory and imaging tests to identify the cause of symptoms. It appears that there could be a financial incentive for hospital systems to specifically encourage early cooperation and planning between cardiology and oncology teams regarding the timing and choice of cancer therapies and coronary revascularization decisions. Our data support the idea that cancer patients could benefit from cardiovascular evaluation and revascularization from such cardio-oncology teams.

This study does have notable limitations which indicate the results should be interpreted cautiously. This is a non-randomized study without longitudinal follow-up that relies upon accuracy of ICD10 coding by providers (i.e., coronary artery disease burden, prior detailed cancer treatment regiments, and overall vs. cardiovascular specific mortality) and a selection bias is possible. By utilizing a large nationally representative dataset and propensity score and machine learning supported analyses with aggressive regression model performance optimization, we attempted to minimize the impact of such limitations and produce the most robust results possible on the association between PCI outcomes and cancer.

## Conclusions

This nationally representative multicenter comprehensive analysis of inpatient mortality and total costs of PCI in all eligible hospitalized patients with and without cancer (including sub-group analysis by CAD, cancer by primary organ site, active vs. prior cancer, and ACS) suggests a significant and independent inpatient mortality and cost benefit for PCI vs. medical management particularly for cancer patients. As there is a unique cancer and coronary artery disease interaction, certain cancer types have a more pronounced mortality benefit compared to others. This study also suggests that PCI was considered in cancer patients regardless of their primary malignancy type, active or prior malignancy status, and ACS status and did not suggest a significant increase in LOS or cost. Our analysis may support future randomized trials to assess the safety and optimal clinical application of coronary revascularization of onco-cardiology patients with both CAD and cancer, while possibly highlighting the current utility of multi-disciplinary teams for this growing and complex patient population.

## Data Availability Statement

The data analyzed in this study is subject to the following licenses/restrictions: The dataset is available for purchase through the United States Agency for Healthcare Research and Quality Healthcare Cost and Utilization Project (HCUP). Requests to access these datasets should be directed to HCUP, HCUPDistributor@ahrq.gov.

## Ethics Statement

The studies involving human participants were reviewed and approved by MD Anderson. Written informed consent for participation was not required for this study in accordance with the national legislation and the institutional requirements.

## Author Contributions

DM created the study design and analyzed the data. DM and SL drafted the manuscript. DM, SL, SP, NP, KC, KM, JL-M, MM, and CI interpreted the data, revised the manuscript, and consented to its publication. All authors contributed to the article and approved the submitted version.

## Conflict of Interest

The authors declare that the research was conducted in the absence of any commercial or financial relationships that could be construed as a potential conflict of interest.
